# The Manchester Royal Infirmary

**Published:** 1902-08-09

**Authors:** 


### The Institutional Workshop.

### THE MANCHESTER ROYAL INFIRMARY.

At a recent special meeting of the Trustees of the Manchester Royal Infirmary, the proposals of the Board of Management for the rebuilding of the Infirmary on its present site in Piccadilly were emphatically rejected, the majority against the acceptance of the plans being estimated to be in the proportion of two to one. A poll of the Trustees was, however, demanded, and the result, which was announced on August 5th, was even more decisive, the voting being nearly at the rate of three to one against the proposal.

The plans which have now been rejected provide for a hospital on a modified pavilion plan with about 450 beds. That the plans are bold and ingenious may well be allowed, but that they are in any way to be accepted as representing the present position in hospital construction cannot be admitted for a moment.

Their primary and radical defect is that the buildings are far too closely crowded together. True, the pavilion plan of construction is followed so far as this, that the principal wards have windows on both sides, but seeing that one side of each pavilion looks into a courtyard, the entrance of which is less than half as wide as the buildings are high, the principal advantages of this mode of construction cannot be said to have been obtained.

The site towards Piccadilly from railing to railing is nearly 630 feet long, and of this frontage a length of 390 feet or thereabouts is occupied by the buildings. The back part of the site, however, which is considerably narrower, is practically covered.

In the centre of the Piccadilly frontage stands the Nurses' Home, a handsome building having a ground floor and five floors above. This is an entirely separate block, connected however with the rest of the hospital by means of a corridor. Behind the Nurses' Home, and approached by a carriage drive, by which this home is surrounded, is the main entrance to the infirmary, leading at once into the casualty department, with surgeries, a series of examination rooms, and two small casualty wards holding two beds each. These are curious little rooms about 12 feet by 20, lighted by windows at one end only, which windows face a lofty blank wall which must be at least 70 feet high.

Behind the casualty department is the administration with offices, board-room, etc., coming nearly up to the boundary of the site at the back, and having on its left side the out-patient department, and on its right, but entirely separated from it, the pathological department.

Between the casualty and the general administration departments is a corridor running right and left and leading to the wards and operating theatres which are symmetrically arranged on each side. This corridor terminates at each end in a circular twenty-bedded ward. These wards come nearly to the edge of the site at each side. Above each of these wards are four others of equal size and from the corridor leading to each of these five-story towers of circular wards there projects forwards towards the Piccadilly frontage a long pavilion, six floors high, providing on four of its floors large wards for patients, while the ground floors and the top story are used partly as wards and partly for lecture theatre, chapel and other purposes. Thus, leaving out of consideration the out-patient department, the new buildings will consist of four great blocks—a nurses' home of six floors in front, an administration block behind, and on each side a pavilion of six floors, with, attached to its outer side, a tower of five floors of circular wards, each pavilion with its annexe containing upwards of 200 beds. We will not now criticise minutiæ, indeed there is much in them that has evidently been well considered, but we would point out as objections to the scheme: first, the height of the buildings in relation to their distance from one another; second, the closeness of some of the buildings to the streets—streets which are themselves so narrow that they must very shortly be widened; third, the noisiness of these streets; and, lastly, the fact that the plan is a complete one, occupying every portion of the land and allowing of no expansion and of but little alteration in accordance with the demands of advancing knowledge and new discoveries.

So far as mere expansion is concerned this objection may not be of much importance, for a hospital of between four and five hundred beds is probably large enough as a unit, and it may be better, when more accommodation is required, to multiply the units than to enlarge them. But we live in days when medical science is advancing by leaps and bounds, when every year sees fresh developments, and when a hospital without any unallotted space which can be turned to new purposes is terribly hampered in its work, and although, if no other alternative were available, the Manchester people would be amply justified in accepting the plan which has been adopted by the board of management, the fact that an alternative does in their case exist entirely alters the position. Within about a mile of the existing site there is another (the Stanley Grove site), the free use of which, it is said, is open to the governors. This site is larger, quieter, nearer to the Owens College, and situated in a more open neighbourhood than the site at Piccadilly, and has, in addition, the undoubted advantage so far as the patients are concerned, that the hospital could be erected and completed without disturbing the present building, whereas if the new hospital is to be built upon the old site, not only will the expense be greatly increased by the necessity of carrying out the work in sections, and of providing temporary accommodation for the patients, but during the whole course of construction, which is estimated to occupy six or seven years, the administration of the hospital will have to be carried on under great disadvantages, and the patients will have to live in a state of constant turmoil.

As regards the objection raised against the Stanley Grove site on the ground of distacne we do not think much of it. Manchester is now so large a place that of necessity the hospital, wherever it may be placed, will be a considerable distance from a large proportion of the population, and with the steady development of the electric tramway system which is taking place, there can be but little doubt that long before the new hospital is finished the Stanley Grove site will be more accessible to the majority of Manchester people than the Royal Infirmary has ever been in times gone by.

Looking at the question from the outside one cannot but feel that in arriving at their decision to rebuild upon the old site, the board of management have been largely swayed by sentiment, by a desire to make no break in the old traditions, and to see in the future as in the past the very centre of this great money-making city occupied by the greatest monument of its charity. With all this everyone will sympathise. But when one considers the crowded streets, the perpetual turmoil, the fact that everything points to Piccadilly being in the future far more than in the past the very centre and crossing place of all the traffic of the city, a place without peace or rest from one end of the year to the other, and the certainty so far as one can forsee the future at all, that before long the demand for far wider streets in this great centre of the tramway system will become imperative, one hesitates to make it also the great centre where the sick are to be brought together for treatment not only from all Manchester but from all Lancashire; and when, in addition to this, one is told that within a short distance a far larger site can be had for nothing, while the present site, according to all estimates, may be sold for so large a sum as to go far towards the building of the new hospital, one is driven still more to the conclusion that it will be far better to go elsewhere. We heartily congratulate the people of Manchester upon the result of the poll.

